# Effects of Human Rights Sensitivity on Empathy and Working Relationship in Mental Health Social Workers: Using Vignettes of Schizophrenia and Alcoholism

**DOI:** 10.3390/ijerph19148668

**Published:** 2022-07-16

**Authors:** Minhwa Lee, Mikyung Seo

**Affiliations:** 1 Department of Social Welfare, Mokpo National University, Muan 58554, Korea; mhsw2020@mnu.ac.kr; 2 Department of Social Welfare, Gyeongsang National University, Jinju 52828, Korea

**Keywords:** empathy, working relationship, human rights sensitivity, moderating effect, vignettes, social worker in mental health

## Abstract

Human rights sensitivity (HRS) is essential for social workers advocating for and providing services to people with mental illness. In this study, the authors employed vignettes of two chronic mental illnesses—schizophrenia and alcoholism—to analyze the moderating effect of HRS on association between empathy and working relationship by hierarchical regression analysis. In total 245 social workers in mental health (M *age* = 36.44, SD = 6.61, male 22.0%, female 78.0%) participated in the study. Differences were found in empathy levels and working relationships in schizophrenia and alcoholism vignettes. Levels of empathy, intrinsic helping and emotional support (behavioral dimension), client respect, and emotional relatedness as well as respect and acceptance in working relationships were significantly higher for schizophrenia than for the alcoholism vignette. Further, empathy and HRS significantly predicted the quality of working relationships in schizophrenia and alcoholism vignettes. Levels for positive work relationships increased with empathy and HRS. The effects of empathy on working relationship were augmented among social workers with a high level of HRS only in the vignette of schizophrenia. Based on these results, the authors emphasize the importance of HRS and propose strategies to enhance it.

## 1. Introduction

Social workers are human-rights-oriented professionals who respect human dignity and worth. They are dedicated to guaranteeing civil liberty. As social workers strive to ensure that the socioeconomically vulnerable populations have a healthy quality of life, they are sensitive to human rights issues. Social workers in the field of mental health advocate for the rights of persons with mental illness and their families and help them realize their civil liberties within the community. Therefore, human rights sensitivity (HRS) is especially important for these professionals. However, persons with mental illness are a vulnerable group whose civil liberties have not been adequately protected due to involuntary hospitalization for decades. They are thought to lack insight, and this is often used to justify restricting their individual liberties to protect them or others from harm. Often, the view of paternalism—temporarily limiting the civil liberty of persons with mental illness who lack the insight to complement their incompetence—is supported [1,2]. However, studies [3,4,5] argue that limiting the rights of persons with mental illness does not help in the long-term improvement of their clinical symptoms and social functioning. Instead, it diminishes treatment adherence and impairs therapeutic relationships. Researchers who support this argument refute paternalism and contend that limiting civil liberty seriously hinders the recovery of persons with mental illness.

Not only are the rights of persons with mental illness violated during admission and stay at mental institutions, but their civil liberty is limited even after returning to the community, due to social stigma. The involuntary admission rate in Korea remains high at 33.6% compared with that in developed countries (20%) [6,7]; owing to social stigma, the public agrees to limiting the driving, employment, and residency of persons with mental illness [8]. Mental health care providers perform two contradictory roles: limiting the rights of persons with mental illness during the treatment process and advocating their rights within the community. They function as both human rights violators and advocates [9]. Recognizing this problem, the Mental Health Improvement and Support of Social Welfare to Persons with Mental Illness Act, revised in 2019, obliges service providers to educate themselves about human rights to enhance their HRS.

Social workers aim to improve the quality of life of persons with mental illness by connecting them to resources, securing their social networks, and training them in various necessary skills. As they are required to advocate for the rights of persons with mental illness against discrimination and exclusion from the community and provide tailored services that meet their needs, social workers’ HRS is especially important for protecting the rights of persons with mental illness.

HRS is the process of recognizing one’s influence on the welfare of others in human-rights-related issues and situations [10]. Specifically, it refers to the process of perceiving and interpreting a particular situation as human-rights-related, considering the available choices, the influence of each choice on the involved persons, and assuming responsibility for resolving the human rights problem [10]. Low HRS among mental health workers is indicative of their greater social distance from persons with mental illness and higher recognition of the need to restrict rights [11], along with stronger support for authoritarianism and social restrictiveness [12].

HRS, an individual’s moral attribute, is closely linked to empathy, which involves recognizing the perspectives of the client—a vulnerable group—and emotionally responding to their affective experiences [13,14]. Empathy, the ability to understand the views of others without sharing their experience and showing concern for them, is strongly associated with HRS, which is dedicated to advocating for others’ rights and assuming responsibility for their own roles [13,14]. In social welfare, empathy is the ability to accurately identify the client’s emotions and convey them, and it is one of the most important attributes in social workers [15]. Empathy in social welfare is a multidimensional construct that encompasses affective and cognitive responses as well as conscious decision-making to take empathic action [16,17]. It is a key factor for improving working relationships (WRs), and all therapeutic models highlight its significance. Lee and Seo [15] showed that empathy is a major predictor of positive WRs by analyzing the effects of empathy for persons with mental illness on WRs of case managers in the public sector across vignette types.

These studies establish that HRS, a characteristic of social workers in mental health, strengthens the effects of empathy in building a positive relationship with persons with mental illness. It should be noted that previous studies found it difficult to suggest strategies to improve the relationship between variables since they focused on the correlation between empathy and WR. Therefore, this study aimed to find a strategy through HRS improvement. The authors predicted that high HRS contributes to bolstering the effect of empathy for persons with mental illness on improving the WR. Therefore, this study analyzed the moderating effect of HRS on the relationship between empathy and WR in schizophrenia and alcoholism vignettes to examine the significance of HRS for social workers in mental health. The vignette approach was used because a broader consideration, such as “client” or “persons with mental illness”, would hinder pinpointing the disability type considered by the participants in their responses. This methodology has been used in recent years for measuring attitudes, emotional responses, and social distance toward persons with mental illness [18,19].

## 2. Materials and Methods

### 2.1. Participants

A total of 245 Korean social workers in mental health, who work in mental health facilities (e.g., community mental health centers, psychiatric rehabilitation centers, and mental hospitals) and provide their clients with various services and interventions between the ages of 20 and 59, were recruited through convenience sampling. Prior to the survey, written informed consent was obtained from all the participants. They comprised 54 males (22.0%) and 191 females (78.0%); their mean age was 36.44 years (SD = 6.61), with 36 participants between 20 and 29 (14.7%), 125 between 30 and 39 (51.0%), 73 between 40 and 49 (29.8%), and 11 between 50 and 59 (4.5%) years. Their mean mental health career was 103.73 (SD = 67.66) months long (Table 1).

Participants were randomly presented with schizophrenia and alcoholism vignettes. In total, 123 participants (50.2%) responded to the schizophrenia vignette, and 122 (49.8%) responded to the alcoholism vignette. Differences in the demographic factors and HRS between the two vignette groups were analyzed with the chi-squared test and independent *t*-test. Gender (χ^2^ = 0.793, *p* = 0.373), age (t = 1.122, *p* = 0.263), career (t = 1.615, *p* = 0.108), and HRS (t = −1.146, *p* = 0.253) did not significantly differ between the two groups.

### 2.2. Measure

#### 2.2.1. Human Rights Sensitivity

HRS is the psychological process of perceiving and interpreting a situation to be human-rights-related, understanding the effects of actions on other people, and assuming responsibility for resolving the situation [10]. In this study, HRS was measured using the scale developed by the National Human Rights Commission of Korea [10], which is based on the scale developed by Volker [20]. This scale rates three factors: situation perception (SP), consequence perception (CP), and responsibility perception (RP), using six items for each of the 10 episodes presented (60 items total). Each factor is rated using two items, one relevant to HRS and one not relevant to HRS. A score is assigned to responses where the item relevant to HRS is rated higher than the item irrelevant to HRS, and the total score is calculated by adding these scores. Each item is rated on a five-point Likert scale (1 = not important at all, 5 = very important), and the total score ranges from 0 to 150. A higher score indicates greater HRS. The Cronbach’s α of the HRS scale is 0.842.

#### 2.2.2. Vignettes

Empathy and WR were assessed using vignettes. Vignettes were designed to describe schizophrenia and alcoholism, two common chronic mental illnesses encountered by mental health service providers. These vignettes were used in previous studies for mental illnesses [21,22,23] and comprise clinical symptoms that are diagnosed based on the fifth edition of the Diagnostic and Statistical Manual of Mental Disorders (DSM-5). The main characters in these vignettes are male participants in their late 40s from a similar economic background. The vignettes include content about characteristic behaviors and symptoms for each disease, but do not present the specific treatment history or diagnosis. The main characters’ gender is set equally among the vignettes because social workers’ rating of the severity of a problem may differ based on the gender of the character, despite them facing similar difficulties [24]. Further, the authors did not present them a diagnosis because any bias could hinder an accurate response assessment of behavioral features [25,26]. Vignettes were assigned randomly to the participants, who were asked to answer the questions on empathy and WR, by imagining they had been assigned the corresponding case in practice.

#### 2.2.3. Empathy

Empathy is “the act of perceiving, understanding, experiencing, and responding to emotional state and idea of another person” [16]. In this study, it was measured using the Korean version of the Empathy Scale for Social Workers (K-ESSW), a Korean-translated and validated version [27] of the ESSW developed by King and Holosko [28]. The 21-item K-ESSW comprises three factors: compassionate contextual assessment (CCA), accepting and attentive collaborative inquiry (ACI), and intrinsic help and emotional support (IHS). CCA, the cognitive aspect of empathy, consists of 10 items for accurately recognizing the client’s perspective, and ACI, the affective version of empathy, consists of five items primarily about trust and bonding between a service provider and a client. The IHS, the behavioral aspect of empathy, consists of six items dealing with protection and altruism. Each item is rated on a five-point Likert scale (1 = strongly disagree, 5 = strongly agree). A higher score indicates a higher level of empathy. The Cronbach’s α of the scale is 0.851.

#### 2.2.4. Working Relationship

WR refers to a relationship between a worker and a client in which both parties collaborate, based on mutual trust, to resolve the client’s needs and problems [29,30]. WR can be understood as individually perceived experiences [31]. In this study, WR was measured using the 21-item Korean version of the Working Relationship Scale for Case Managers, developed, and validated by Kwon [31], which consists of three factors: collaborative relationship (CR), client respect and emotional relatedness (ER), and professional contribution (PC). CR comprises 10 items concerning collaborative work between a case manager and a client, and ER comprises seven items concerning client respect and acceptance. PC comprises four items on trust in case manager’s expertise and expectations for positive outcomes of intervention. Each item is rated on a five-point Likert scale (1 = strongly disagree, 5 = strongly agree); a higher score indicates a greater level of positive WR. The Cronbach’s α of the scale is 0.898.

### 2.3. Data Analysis

The collected data were analyzed using the SPSS version 27.0 for Windows (IBM Corp., Armonk, NY, USA) program. The normality of all major parameters was assessed, and the reliability of the scales was analyzed. First, participants’ sociodemographic characteristics were analyzed through frequency statistics and descriptive statistics. Second, differences in participants’ characteristics by vignette type were analyzed using the chi-squared test, and differences in the mean scores for major variables by vignette type were analyzed using an independent-sample *t*-test. Third, relationships among major variables were analyzed using Pearson’s correlation. Fourth, the effects of HRS on the relationship between empathy and WR were analyzed through hierarchical regression analysis. First, mean-centered empathy and HRS were used to resolve the multicollinearity problem. Then, a three-step hierarchical regression analysis was performed. In step 1, the control variables—demographic characteristics, gender, age, and length of career— as well as dependent variables (i.e., WR) were entered. In step 2, the control variables, independent variable (i.e., empathy), moderator variable (i.e., HRS), and dependent variable were analyzed. In step 3, the control variables, independent variable moderator variable, interaction term (i.e., empathy × HRS), and dependent variable were analyzed. In step 3, the moderating effect was evaluated by analyzing the significance of the change in *R*^2^. The moderator was found to be significant, a graph was plotted, and simple slope analysis was performed.

## 3. Result

### 3.1. Differences in Empathy and WR between Vignettes

The mean differences of empathy and WR between vignettes were analyzed using an independent-sample *t*-test (Table 2). The total empathy score was significantly higher in the schizophrenia vignette (M = 4.09, SD = 0.31) than the alcoholism vignette (M = 3.98, SD = 0.28). By subscale, the IHS score (behavioral dimension) was significantly higher in the schizophrenia vignette (M = 3.71, SD = 0.47) than the alcoholism vignette (M = 3.51, SD = 0.39). Regarding WR, only the ER score (i.e., respect for and acceptance of client) significantly differed between the vignettes. The results showed that the mean score was higher for the schizophrenia vignette (M = 4.33, SD = 0.41) than for the alcoholism vignette (M = 4.17, SD = 0.39). The total WR score, and other subscale scores were similar for both the vignettes, without significant differences.

### 3.2. Correlations among Variables

Table 3 shows the correlation analysis of the study variables. Empathy was found to have a statistically significant positive correlation with HRS (r = 0.206) and WR (r = 0.377). HRS had a statistically significant positive correlation with WR (r = 0.286). The correlations among the variables were above r < 0.4, confirming the absence of multicollinearity.

### 3.3. Moderating Effect of HRS on the Association between Empathy and WR

Hierarchical regression analysis was performed to examine the effects of HRS on the association between empathy and WR. To rule out multi-collinearity, variance inflation factor values of the variables were analyzed. In the regression model, these values were found to be less than three, and hence, multi-collinearity was ruled out.

Table 4 shows the results for the schizophrenia vignette. The effects of control variables were analyzed in model 1, and gender, age, and length of career were not statistically significantly associated with WR. In model 2, empathy (independent variable) and HRS (moderator) were additionally entered. This model accounted for 24.2% of the variance in WR (F = 7.283, *p* < 0.001). Empathy (β = 0.383, t = 4.425, *p* < 0.001) and HRS (β = 0.213, t = 2.437, *p* < 0.05) were found to have a significant association with the measures of WR. It was found that social workers with higher levels of empathy and HRS build higher positive WR. Finally, to examine the moderating role of HRS, the interaction term (empathy × HRS) was computed. Model 3 with the interaction term added to the control variables, empathy, and HRS accounted for 29.7% of the variance in WR (F = 7.939, *p* < 0.001). The results showed that *R*^2^ increased by 5.4%, and the change was statistically significant at *p* < 0.01. The interaction term additionally explains 5.4% of the variance of WR, suggesting that HRS has a significant moderating effect. The coefficient value for the interaction term was positive, that is, the interaction between empathy and HRS was found to be positively associated with WR (β = 0.251, t = 2.957, *p* < 0.01, 95% CI = [0.926, 3.586]).

Furthermore, to understand the interactions within the moderation, simple slope comparisons between high (+1 SD), medium, and low (−1 SD) values of the moderators were performed. Simple slope difference tests are mostly used to determine three-way interaction within moderated multiple regression models [32] and assess the impact of extreme values [33].

**Figure 1:**
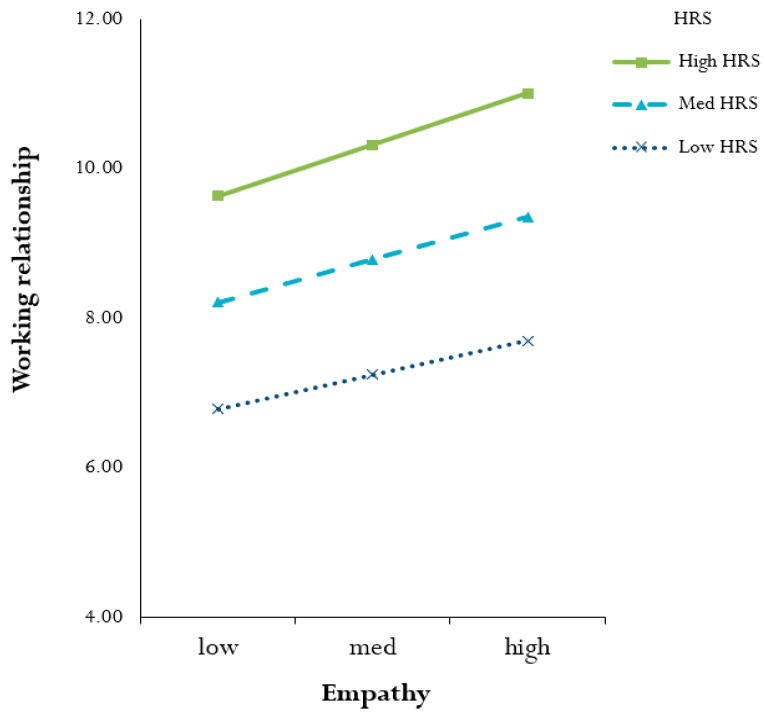
Simple slopes for the moderating effect of HRS on the relationship between empathy and WR (high HRS: slope = 2.16, t = 3.807, *p* < 0.001; med HRS: slope = 1.80, t = 4.003, *p* < 0.001; low HRS: slope = 1.44, t = 4.309, *p* < 0.001).

Figure 1 shows the simple slope plot for different levels of HRS. The linear trend for WR was found to be statistically significant and positive at level 0.001. All the slopes were positive, although the slope for high HRS was steeper than that for others. A single standard deviation increase in empathy was found to be associated with a higher positive WR at a high HRS. It was observed that HRS strengthened the positive relationship between empathy and WR.

Table 5 shows the results of the hierarchical regression performed to examine the interaction effects of HRS in the alcoholism vignette. The effects of control variables were analyzed in model 1, and gender, age, and length of career were not significantly associated with WR. In model 2, empathy and HRS were additionally entered. This model accounted for an average of 17% of the variance in WR (F = 4.717, *p* < 0.01). Empathy (β = 0.238, t = 2.688, *p* < 0.01) and HRS (β = 0.289, t = 3.347, *p* < 0.01) were found to have a significant association with measures of WR. As with the schizophrenia vignette, the degree of positive WR increased with empathy and HRS in the alcoholism vignette. In model 3, the interaction term (empathy × HRS) was added to evaluate the moderating effect of HRS. The interaction term, along with the control variables, empathy, and HRS, accounted for an average of 17.2% of the variance in WR (F = 3.938, *p* < 0.01). Results showed that the *R*^2^ increased by 0.1% as compared to model 2, but the change was not statistically significant. It was also found that the interaction term was not statistically significant, indicating that HRS did not have a significant moderating effect.

## 4. Discussion

This study highlighted the importance of HRS in mental health social workers who advocate for the rights of persons with mental illness. It examined the effects of HRS on empathy and WR in 245 social workers in mental health facilities. Vignettes of two common chronic mental illnesses—schizophrenia and alcoholism—were employed to analyze the moderating effect of HRS on the association between empathy and WR. The key findings of this study are outlined below.

First, the authors analyzed the differences in empathy and WR between the schizophrenia and alcoholism vignettes. Results showed that the scores of total empathy, IHS (behavioral dimension of empathy), and ER (respect and acceptance in WR) were higher for the schizophrenia vignette than the alcoholism vignette. However, there were no significant differences in scores for other factors. Social workers in mental health must respect, accept, and advocate for the interests of all clients without discrimination. Nevertheless, their level of empathy and a few dimensions of positive WR were found to differ significantly between the two frequently encountered mental health conditions (schizophrenia and alcoholism). Social workers were found to have higher empathy, respect, and acceptance for patients with schizophrenia than for alcoholics. This suggests that prejudice and discrimination also contribute to the difference in workers’ responses to each vignette. Many studies that compared the social stigma against schizophrenia and alcoholism [21,22,34,35,36,37,38] reported that the public perceives alcoholism to be more dangerous than schizophrenia. They want to greater socially distance themselves from alcoholics and experience a greater amount of negative emotion such as anger toward them. According to researchers, this is because people attribute alcoholism to controllable factors, such as lack of willpower and habit problems. People attribute schizophrenia to uncontrollable, biological factors, while they attribute alcoholism to personal habits or willpower, thereby displaying greater negative responses toward alcoholism. Although social workers in mental health are provided a scientific understanding of each diagnosis through professional education, these results suggest that they may be influenced by a prejudice acquired through socialization over a long time.

Second, the authors analyzed the effects of HRS on empathy and WR between the vignettes. The results showed that empathy and HRS significantly predicted WR in both the vignettes. It was found that positive WR increased with the increase in empathy and HRS. However, HRS significantly moderated the association between empathy and WR only in the schizophrenia vignette and not in the alcoholism vignette. This suggests that the effects of empathy on WR were augmented among social workers with a high level of HRS only in the case of schizophrenia. In the alcoholism vignette, having a high HRS did not significantly increase the effect of empathy on WR. This is similar to the results reported by Lee and Seo [15], where the effect of empathy on WR is greater in schizophrenia than in alcoholism. However, the said study did not examine the moderating effect of HRS. Taken together, these results show that HRS based on individualizing moral foundation does not have a consistent effect across all types of cases in professional practice. Even though HRS is closely linked to empathy [13,14] and empathy is a key predictor of positive WR [39,40,41], it does not have a strong influence on those in the alcoholism group. Silva et al. [42] reported that service providers’ views of ethical issues pertaining to threats were bound to differ, concluding, “there is no one size fits all”. Thus, a similar conclusion can be drawn in the present study. Even if a social worker has a high HRS, establishing a positive relationship with the client by empathizing with them is not irrelevant to their attitudes and emotions toward the case. As previously mentioned, alcoholism is perceived as more threatening and tagged with greater negative emotions than schizophrenia, so HRS is anticipated to have a lower influence on the association between empathy and WR.

## 5. Conclusions

Based on these results, the authors present strategies to improve HRS of mental health service providers. First, the contents of education programs that aim to improve HRS among service providers should be reviewed. While a focus on imparting an understanding of the basic rights is important, ensuring that human rights education has a positive influence on the delivery of mental health services is crucial, as that can have a direct impact on protecting the rights of persons with mental illness. These education programs should be structured such that workers learn to empathize with persons with mental illness and foster a positive WR with them. Recent studies have proposed the use of virtual-reality-based programs for boosting empathy and HRS [14]. Hence, strategies to provide an opportunity for individuals to experience mental illness in virtual reality to enhance their empathy and HRS could be considered.

Second, efforts to improve the perception of alcoholism across all mental health service providers, including social workers, and the public are necessary. While schizophrenia is still perceived negatively in society, alcoholism is considered even worse. Owing to this negative social perception, patients suffering from the condition are hesitant to disclose their problems and seek help. In the end, they are placed in the treatment system only after the situation worsens to the point of requiring emergency intervention. This, in turn, hinders service providers from empathizing and fostering a positive WR with them, further aggravating their negative perceptions. Thus, tailored anti-stigma strategies for alcoholism are needed. This is consistent with the arguments of most researchers who studied social stigma using the vignette approach [18,36,38,43,44].

### Limitations

This study focused on social workers in mental health and showed that the effects of HRS on empathy and WR differ between schizophrenia and alcoholism vignettes. Nevertheless, the study has certain limitations. First, it is difficult to generalize the influence of social workers’ HRS on all forms of mental illnesses solely based on those findings, as the authors focused only on schizophrenia and alcoholism. It is suggested that depression, a highly prevalent mental illness, be also included in future studies. Depression is a globally common disease with a prevalence of 5.0% among adults (5.7% in adults aged 60 years and over) [45], and in Korea, the prevalence of mood disorders, including depression, is 5.3%, compared to 25.4% of all mental disorders [46]. Second, social workers have a high level of HRS compared with other mental health service providers [9]. As the findings of this study pertain to the influence of HRS of this specific population, generalization to other mental health providers is limited. A multidisciplinary approach inclusive of other types of providers is required to protect the rights of persons with mental illness. Third, WR refers to a relationship between a social worker and a client. This study measured the level of WR based on the subjective perception of the social workers in the vignettes. Therefore, it cannot be assured that WR in the real world will be similar to the results of this study, and thus the best approach is to evaluate the WR for a social worker and a client in the real world. However, there are limitations that cannot be directly assessed due to various practical problems, e.g., confidentiality and client recruitment with written consent. Fourth, discussion of findings and consideration of the literature were hindered by the scarcity of studies focusing on the relationship between HRS and empathy in the field of mental health.

## Figures and Tables

**Table 1:** Comparison of sociodemographic characteristics and HRS between vignettes.

Variable	Vignettes				Overall		χ^2^/t (*p*)
	Schizophrenia		Alcoholism				
	N	%	N	%	N	%	
	123	50.2	122	49.8	245	100.0	
Gender							
Male	30	12.2	24	9.8	54	22.0	0.793 (0.373)
Female	93	38.0	98	40.0	191	78.0	
Age (1)	36.91 (±7.17)		35.96 (±5.99)		36.44 (±6.61)		1.122 (0.263)
20–29	17	6.9	19	7.8	36	14.7	5.077 (0.166)
30–39	59	24.1	66	26.9	125	51.0	
40–49	38	15.5	35	14.3	73	29.8	
50–59	9	3.7	2	0.8	11	4.5	
Career (2)	110.74 (±69.85)		96.77 (±64.96)		103.73 (±67.66)		1.615 (0.108)
HRS	88.19 (±25.55)		91.92 (±25.38)		90.05 (±25.48)		−1.146 (0.253)
SP	28.82 (±9.41)		29.86 (±9.27)		29.33 (±9.34)		−0.870 (0.385)
CP	28.99 (±9.42)		30.54 (±9.87)		29.76 (±9.66)		−1.256 (0.210)
RP	30.38 (±8.84)		31.52 (±7.90)		30.95 (±8.38)		−1.066 (0.287)

(1) age: years; (2) career: months; HRS: human rights sensitivity; SP: situation perception; CP: consequence perception; RP: responsibility perception.

**Table 2:** Comparison of mean scores of empathy and WR between vignettes.

Variable	Schizophrenia	Alcoholism	*t*-Test	*p*-Value
Empathy	4.09 (±0.31)	3.98 (±0.28)	2.681	0.008
CCA	4.26 (±0.36)	4.19 (±0.35)	1.517	0.131
ACI	4.20 (±0.39)	4.13 (±0.43)	1.266	0.207
IHS	3.71 (±0.47)	3.51 (±0.39)	3.419	0.001
Working Relationship	3.73 (±0.41)	3.67 (±0.38)	1.165	0.245
CR	3.29 (±0.62)	3.29 (±0.51)	−0.033	0.974
ER	4.33 (±0.41)	4.17 (±0.39)	3.087	0.002
PC	3.78 (±0.49)	3.74 (±0.48)	0.652	0.515

CCA: compassionate contextual assessment; ACI: accepting and attentive collaborative inquiry; IHS: intrinsic help and emotional support; CR: cooperative relationship; ER: client respect and emotional relatedness; PC: professional contribution.

**Table 3:** Correlations among the study variables.

	1	2	3
1. Empathy	-		
2. Human Rights Sensitivity	0.206 **	-	
3. Working Relationship	0.377 **	0.286 **	-

** *p* < 0.01.

**Table 4:** Impacts of HRS on empathy and WR with SPR vignette.

Independence Variable	Working Relationship					
	Model 1		Model 2		Model 3	
	β	*p*	β	*p*	β	*p*
Control Variables						
Gender	0.045	0.627	−0.035	0.677	−0.069	0.401
Age	0.194	0.205	0.038	0.787	−0.040	0.768
Career	−0.084	0.581	0.029	0.836	0.129	0.351
Main Effect						
Empathy			0.383	<0.001	0.436	<0.001
HRS			0.213	0.016	0.179	0.037
Interaction Effects						
Empathy × HRS					0.251	0.004
Overall Model						
F value	0.859		7.283 ***		7.939 ***	
Δ*R*^2^	0.022		0.220 ***		0.054 **	
*R* ^2^	0.022		0.242		0.297	
Adj *R*^2^	−0.004		0.209		0.259	

SPR: schizophrenia; HRS: human rights sensitivity. Dummy: gender (1 = male participant); ** *p* < 0.01, *** *p* < 0.001.

**Table 5:** Impact of HRS on empathy and WR with ALC vignette.

Independence Variable	Working Relationship					
	Model 1		Model 2		Model 3	
	β	*p*	β	*p*	β	*p*
Control Variable						
Gender	0.067	0.470	0.039	0.654	0.041	0.641
Age	−0.130	0.347	−0.077	0.552	−0.068	0.606
Career	0.106	0.445	0.040	0.759	0.029	0.826
Main Effect						
Empathy			0.238	0.008	0.230	0.013
HRS			0.289	0.001	0.283	0.002
Interaction Effects						
Empathy × HRS					0.041	0.652
Overall Model						
F value	0.444		4.717 **		3.938 **	
Δ*R*^2^	0.011		0.159 ***		0.001	
*R* ^2^	0.011		0.170		0.172	
Adj *R*^2^	−0.014		0.134		0.128	

ALC: alcoholism; HRS: human rights sensitivity. Dummy: gender (1 = male participant); ** *p* < 0.01, *** *p* < 0.001.
